# Clinical Characteristics and Outcomes of Hospitalized Patients With COVID-19 and Type 2 Diabetes in Vietnam: A Retrospective Study

**DOI:** 10.7759/cureus.108011

**Published:** 2026-04-30

**Authors:** Thuy Anh T Huynh, Chau Le Buu, Bao An H Nguyen, Trinh T Tran, Khanh Nguyen, Phuong Trang Van, Thu Thao Nguyen, Tien Thanh Nguyen, Vinh Khoa T Ton, Diem T Le

**Affiliations:** 1 University of Health Sciences, Vietnam National University Ho Chi Minh City, Ho Chi Minh City, VNM; 2 Infectious Diseases Department D, Hospital for Tropical Diseases, Ho Chi Minh City, VNM; 3 Infectious Diseases, University of Medicine and Pharmacy at Ho Chi Minh City, Ho Chi Minh City, VNM; 4 Infectious Diseases Department B, Hospital for Tropical Diseases, Ho Chi Minh City, VNM

**Keywords:** covid-19, diabetes mellitus type 2, disease severity, hospital outcomes, length of stay, mortality, retrospective study, vietnam

## Abstract

Objective: To describe the clinical characteristics and outcomes of hospitalized patients with COVID-19 and diabetes mellitus in Vietnam.

Materials and methods: A retrospective descriptive study was carried out from June 2021 to September 2022 at the Hospital for Tropical Diseases, a tertiary care center in Ho Chi Minh City, Vietnam.

Results: The study included 154 patients with COVID-19 and diabetes mellitus. The mean age was 65 years, and women accounted for 64.3% of the study population. Prior COVID-19 vaccination was documented in 39.6% of patients. The mean duration of illness before hospitalization was 5 ± 3 days (range, one to 15 days). At admission, 71.8% of patients required oxygen support. The most common clinical manifestations were cough, dyspnea, and fever, while chest pain and diarrhea were less frequently observed. Common in-hospital complications included cytokine storm, acute respiratory distress syndrome (ARDS), sepsis, septic shock, ventilator-associated pneumonia, and urinary tract infection. Less frequent complications were ketoacidosis, hyperosmolar hyperglycemic state, and myocardial infarction. Most patients had a previous diagnosis of diabetes, whereas 14.3% were newly diagnosed during hospitalization on the basis of glycated hemoglobin (HbA1c). The mean length of hospital stay was 16 ± 10 days (range, two to 71 days), and the mortality rate was 20%.

Conclusion: Hospitalized patients with COVID-19 and diabetes mellitus had a substantial clinical burden, reflected by frequent oxygen requirements, severe complications, prolonged hospitalization, and considerable mortality. These findings underscore the need for close monitoring and careful metabolic management in this high-risk population.

## Introduction

Diabetes mellitus has been consistently recognized as an important comorbidity associated with poor outcomes in patients with COVID-19. In individuals with diabetes, severe acute respiratory syndrome coronavirus 2 (SARS-CoV-2) infection may further worsen glucose control through systemic inflammation, stress hyperglycemia, and the effects of glucocorticoids commonly used in COVID-19 management. These factors may contribute to hyperglycemia and the identification of previously unrecognized type 2 diabetes mellitus (T2DM) during hospitalization [1].

Previous studies have shown that patients with diabetes have higher mortality rates, ranging from 20% to 28% [2-4], and are more likely to develop severe COVID-19, acute respiratory distress syndrome (ARDS), clinical deterioration, ICU admission, and need for mechanical ventilation [5].

Despite these associations, most available data have been derived from Western populations. Evidence from Southeast Asia, particularly Vietnam, remains limited. Moreover, the clinical characteristics and outcomes of hospitalized patients with COVID-19 and T2DM, including cases newly identified during hospitalization based on glycated hemoglobin (HbA1c), have not been adequately described in the local context. Because HbA1c testing was selectively performed during hospitalization rather than routinely applied to all patients, newly identified cases should be interpreted cautiously.

A clearer understanding of the clinical profile, complications, treatment patterns, and outcomes of these patients is important to support early identification, inpatient glycemic management, and clinical decision-making in tertiary care settings.

Therefore, this retrospective descriptive study was conducted to describe the clinical characteristics and outcomes of hospitalized patients with COVID-19 and T2DM, including both previously diagnosed cases and newly identified cases based on selective HbA1c testing during hospitalization, at the Hospital for Tropical Diseases in Ho Chi Minh City, Vietnam, between June 2021 and September 2022.

## Materials and methods

Study design and setting

This retrospective descriptive study was conducted at the Hospital for Tropical Diseases, Ho Chi Minh City, Vietnam, from June 2021 to September 2022. This retrospective study was approved by the Ethics Committee of the University of Health Sciences, Vietnam National University Ho Chi Minh City (approval no. 211/QĐ-ĐHQG). 

Study population

The study population included patients diagnosed with COVID-19 and T2DM who were admitted to the Hospital for Tropical Diseases during the study period.

Inclusion and exclusion criteria 

Patients were included if they were aged 16 years or older, had a confirmed diagnosis of COVID-19 defined as compatible clinical manifestations and a positive test result for SARS-CoV-2 by polymerase chain reaction (PCR) or antigen detection immunoassay [6], and had T2DM. T2DM was defined as either previously diagnosed and treated T2DM or newly diagnosed T2DM during hospitalization with HbA1c ≥6.5% (48 mmol/mol) [7].

Patients were excluded if they were pregnant or if they had elevated fasting plasma glucose suggestive of diabetes (≥126 mg/dL) but had no previous history of T2DM and lacked confirmatory HbA1c testing during hospitalization, making definitive classification impossible.

Data collection and measurements 

Data were retrospectively extracted from electronic medical records of eligible patients using standardized data collection forms by the research team. The collected data included demographic characteristics, COVID-19 vaccination status, comorbidities, duration of illness before hospitalization, clinical manifestations, mental status, oxygen saturation, and oxygen requirement at admission, laboratory and imaging findings, microbiological results, treatments during hospitalization, complications, length of hospital stay, duration of ICU stay, duration of respiratory support, and clinical outcomes, including recovery and in-hospital mortality.

The extracted data were subsequently reviewed for completeness and consistency by the research team. Missing data were handled according to data availability in the medical records, and analyses were performed using available-case reporting without imputation, consistent with the descriptive nature of the study.

HbA1c testing was not routinely performed for all hospitalized patients during the study period and was mainly requested based on clinical judgment, particularly in patients with suspected previously unrecognized diabetes or poor glycemic control. Newly diagnosed T2DM was identified in patients without a previous history of T2DM who had HbA1c ≥6.5% during hospitalization. Among the 24 patients who underwent HbA1c testing, 22 were newly identified cases, and two had previously known diabetes and underwent HbA1c testing for follow-up. This selective testing may have introduced potential selection bias when interpreting newly identified cases.

Definitions

COVID-19 severity was classified into four levels, namely mild, moderate, severe, and critical, according to the Vietnamese Ministry of Health guideline on diagnosis and treatment of COVID-19 applicable during the study period (Decision No. 2671/QD-BYT). Mild disease was defined as SpO₂ >96% and respiratory rate <20 breaths/minute. Moderate disease was defined as SpO₂ 94%-96%, respiratory rate 20-25 breaths/minute, chest X-ray involvement <50%, or mild COVID-19 in the presence of underlying comorbidities. Severe disease was defined as SpO₂ <94%, respiratory rate >25 breaths/minute, and chest X-ray involvement >50%. Critical disease was defined as respiratory failure requiring endotracheal intubation and invasive mechanical ventilation, shock, or multiple organ failure [8].

ARDS was defined according to the Berlin 2012 criteria [9], including acute onset within seven days; bilateral pulmonary infiltrates on chest X-ray or CT scan not explained by pleural effusion, lobar collapse, atelectasis, or pulmonary nodules; absence of clinical evidence of left atrial hypertension or cardiac failure or fluid overload; and hypoxemia with PaO₂/FiO₂ ≤300 on mechanical ventilation with positive end-expiratory pressure (PEEP) ≥5 cmH₂O. ARDS severity was further classified according to the PaO₂/FiO₂ ratio into three groups: >200, >100 to ≤200, and ≤100.

A cytokine storm in patients with COVID-19 was diagnosed when the lymphocyte count was <1,000 cells/mm³ together with the following criteria: D-dimer >1,000 ng/mL, lactate dehydrogenase (LDH) >300 U/L, and ferritin >500 ng/mL [10]. Patients were considered to have the cytokine storm if they met at least two of the three criteria above or at least one criterion together with C-reactive protein (CRP) >10 mg/dL.

Diabetic ketoacidosis was diagnosed when all three of the following criteria were present: hyperglycemia, usually 350-500 mg/dL (19.5-28.0 mmol/L), although in some cases blood glucose may be only mildly elevated; high anion gap metabolic acidosis; and ketonemia [11].

Acute kidney injury during hospitalization was defined according to the Kidney Disease: Improving Global Outcomes (KDIGO) 2012 criteria as the presence of at least one of the following: an increase in serum creatinine ≥0.3 mg/dL (≥26.5 µmol/L) within 48 hours, an increase in serum creatinine to ≥1.5 times baseline within the previous seven days, or urine output <0.5 mL/kg/hour for six hours [12].

Sample size

All patients who met the eligibility criteria during the study period were consecutively included, and no formal sample size calculation was performed.

Statistical analysis

Baseline patient characteristics were summarized using descriptive statistics. Data entry was performed using Microsoft Excel (Microsoft Corporation, Redmond, Washington, United States) and EpiData version 3.1 (EpiData Association, Odense, Denmark), and statistical analyses were conducted using STATA version 16.0 (StataCorp LLC, College Station, Texas, United States).

Continuous variables were presented as mean ± standard deviation (SD) or median (interquartile range, IQR), as appropriate. Categorical variables were presented as frequencies and percentages.

No univariable or multivariable analyses were performed because this study was designed as a retrospective descriptive study with the primary objective of characterizing clinical features and outcomes. In addition, the relatively limited number of outcome events and incomplete data for several variables limited the feasibility of reliable inferential analyses.

## Results

Clinical characteristics

A total of 154 patients with COVID-19 and diabetes mellitus were included in the study between June 2021 and September 2022. The baseline demographic and clinical characteristics of the study population are presented in Table 1. The study population was predominantly older and female, with 99 patients (64.3%) aged ≥60 years and 99 patients (64.3%) being female. Hypertension (120/154, 77.9%) and overweight/obesity (93/154, 60.4%) were the most common comorbidities. At admission, the mean duration of illness was 5 ± 3 days (range: one to 15). Most patients were alert (139/154, 90.3%), while 15 patients (9.7%) had an altered mental status. Oxygen therapy was required in 107 patients (71.8%). The most common symptoms during hospitalization were cough (136/154, 88.3%), dyspnea (127/154, 82.5%), and fever (103/154, 66.9%). Regarding complications, cytokine storm occurred in 84 patients (54.0%), ARDS in 42 patients (27.3%), and sepsis or septic shock in 37 patients (24.0%).

**Table 1:** Clinical characteristics Data are presented as n (%), mean ± standard deviation (SD), or median (P25–P75), as appropriate. ARDS: acute respiratory distress syndrome; AKI: acute kidney injury

		Characteristics	Frequency (n)	Percentage (%)
Population characteristics				
Age, years, mean ± SD (range)			65 ± 13 (35–101)	
Age group		<60	55	35.7
		≥60	99	64.3
Sex		Male	55	35.7
		Female	99	64.3
COVID-19 vaccination		Yes	61	39.6
		No	31	20.1
		Unknown	62	40.3
Other comorbidities		Hypertension	120	77.9
		Overweight/obesity	93	60.4
		Chronic kidney disease	24	15.6
		Dyslipidemia	22	14.3
		Cardiovascular disease	16	10.4
		Fatty liver disease	7	4.5
		Stroke	5	3.3
Clinical characteristics on admission				
Duration of illness, days, mean ± SD (range) (n=154)			5 ± 3 (1–15)	
Mental status (n=154)	Alert		139	90.3
	Altered mental status		15	9.7
SpO_2_ (n=154)	SpO_2_ ≥ 95%		85	55.2
	SpO_2_ < 95%		69	44.8
Oxygen therapy (n=154)	Yes		107	71.8
	No		47	28.2
Symptoms during hospitalization				
Clinical characteristics	Cough		136	88.3
	Dyspnea		127	82.5
	Fever		103	66.9
	Chest pain		12	7.8
	Diarrhea		10	6.5
Clinical complications	Cytokine storm		84	54.0
	ARDS		42	27.3
	Sepsis/Septic shock		37	24.0
	Ventilator-associated pneumonia		31	20.1
	AKI		26	16.9
	Urinary tract infection		26	16.9
	Diabetic ketoacidosis		15	9.7
	Myocardial infarction		9	5.8
	Hyperosmolar hyperglycemic state		6	3.9

COVID-19 severity and disease progression

Among the 43 patients with mild disease at admission, 12 patients (27.9%) progressed to moderate severity, one patient (2.3%) to severe disease, and five patients (11.6%) to critical illness. Of the 53 patients with moderate disease at admission, three patients (5.7%) progressed to severe disease, and 12 patients (22.6%) to critical condition. Among the 28 patients with severe disease at admission, 18 patients (64.3%) progressed to critical condition. The mean time to disease progression was 8 ± 4 days (range: two to 21 days).

Laboratory characteristics

Laboratory findings are presented in Table 2. The mean blood glucose level at admission was 243 ± 152 mg/dL (range: 46-981), and the mean blood glucose level during hospitalization was 223 ± 55 mg/dL (range: 93-348). Among the 24 patients with available HbA1c measurements, the mean HbA1c was 7.9 ± 1.6%. Markers of inflammation were elevated, with a median CRP of 64 mg/L (IQR: 23-117) and ferritin of 726 ng/mL (IQR: 266-1675). These findings suggest a significant inflammatory response in the study population.

**Table 2:** Laboratory results Data are presented as mean ± standard deviation (SD) or median (P25-P75), as appropriate. Mean blood glucose during hospitalization reflects inpatient glycemic control; therefore, the recommended treatment target of 100-180 mg/dL is presented instead of a conventional laboratory reference range. SD: standard deviation; HbA1c: glycated hemoglobin; APTT: activated partial thromboplastin time; PaO_2_: partial pressure of arterial oxygen; PaCO_2_: partial pressure of arterial carbon dioxide; HCO_3_: bicarbonate; AaDO_2_: alveolar-arterial oxygen gradient; AST: aspartate aminotransferase; ALT: alanine aminotransferase; LDH: lactate dehydrogenase; CRP: C-reactive protein; IL-6: interleukin-6

Laboratory parameter	Value
Blood glucose on admission, mg/dL, mean ± SD (range) (n=154)	243 ± 152 (46–981)
Mean blood glucose during hospitalization, mg/dL, mean ± SD (range) (n=154)	223 ± 55 (93–348)
HbA1c, %, mean ± SD (range) (n=24)	7.9 ± 1.6 (6.2–12.2)
White blood cell count, K/µL (n=154)	8.4 (6.4–11.6)
Platelet count, K/µL (n=154)	213 (157–258)
Prothrombin activity, % (n=48)	86 (54–100)
APTT ratio (n=48)	1.4 (1.1–3.0)
D-dimer, µg/mL (n=139)	0.7 (0.5–1.4)
Fibrinogen, g/L (n=139)	5.4 (4.5–6.6)
pH (n=92)	7.43 (7.38–7.46)
PaO_2_, mmHg (n=92)	89 (75–104)
PaCO_2_, mmHg (n=92)	35 (30–40)
HCO_3_, mEq/L (n=92)	23 (20–25)
AaDO_2_, mmHg (n=92)	250 (120–453)
Arterial lactate, mmol/L (n=84)	2.6 (2.1–4.3)
Creatinine, µmol/L (n=151)	79 (62–107)
AST, U/L (n=148)	36 (25–52)
ALT, U/L (n=148)	30 (21–43)
Total bilirubin, µmol/L (n=36)	10 (7–15)
LDH, U/L (n=39)	361 (269–522)
Ferritin, ng/mL (n=66)	726 (266–1675)
CRP, mg/L (n=108)	64 (23–117)
Procalcitonin, ng/mL (n=30)	0.54 (0.25–3.88)
IL-6, pg/mL (n=60)	24.5 (8–73)
Albumin, g/L (n=46)	29 (25–33)
Troponin I, ng/L (n=68)	31 (10–275)
Potassium, mmol/L (n=153)	4.3 (3.8–4.8)
Sodium, mmol/L (n=153)	129 (125–132)

Imaging characteristics

Imaging findings on admission are summarized in Table 3. Among the 148 patients who underwent chest X-ray, pulmonary infiltration was observed in 133 patients (89.8%), while pleural effusion was present in 21 patients (14.2%). Echocardiography was performed in 46 patients, of whom 39 (84.8%) had valvular regurgitation, and five (10.9%) had pericardial effusion.

**Table 3:** Imaging characteristics upon admission Data are presented as n (%) or mean ± standard deviation (SD), as appropriate.

Imaging modality / Finding	Frequency (n)	Percentage (%)
Chest X-ray (n=148)		
Infiltration	133	89.8
Pleural effusion	21	14.2
Pneumothorax	3	2.0
Atelectasis	1	0.7
Echocardiography (n=46)		
Valvular regurgitation	39	84.8
Pericardial effusion	5	10.9
Ejection fraction, %, mean ± SD (range)	58 ± 8 (32–74)	

Microbiological characteristics

Bronchoalveolar lavage cultures were performed in 35 patients, of whom 22 (62.9%) yielded positive results. The most frequently identified microorganisms were *Acinetobacter baumannii*, *Klebsiella pneumoniae*, *Pseudomonas aeruginosa*, and methicillin-resistant *Staphylococcus aureus*. Blood cultures were obtained from 116 patients, with 16 (13.8%) yielding positive results. Urine cultures were performed in 44 patients, of which 27 (61.4%) were positive.

Treatment characteristics

Treatment characteristics are presented in Table 4. Most patients received anticoagulants (139/154, 90.3%), corticosteroids (138/154, 89.6%), and antibiotics (137/154, 88.9%). Respiratory support was required in 129 patients (83.8%), with invasive mechanical ventilation used in 42 patients (32.5%).

**Table 4:** Treatment characteristics of the study population (n=154) Data are presented as n (%). HFNC: high-flow nasal cannula; NIV: non-invasive ventilation; ECMO: extracorporeal membrane oxygenation

Treatment	Frequency (n)	Percentage (%)
Antibiotics	137	88.9
Anticoagulants	139	90.3
Corticosteroids	138	89.6
Antivirals	70	45.5
Albumin	27	17.5
Antidiabetic medications	131	85.1
Respiratory support	129	83.8
Highest level of respiratory support		
Nasal cannula	50	38.7
Face mask	14	10.8
HFNC	21	16.2
NIV	2	1.5
Invasive mechanical ventilation	42	32.5
Hemofiltration	4	2.6
ECMO	1	0.6

Clinical outcomes

The mean length of hospital stay was 16 ± 10 days (range: two to 71 days). The mean ICU stay was 17 ± 15 days (range: one to 71 days), and the mean duration of respiratory support was 13 ± 12 days (range: one to 71 days). Overall, 31 patients (20.1%) died, while 123 patients (79.9%) recovered.

## Discussion

Clinical characteristics

In this study population, female patients with COVID-19 and T2DM constituted the larger proportion. Similar findings have been reported in several studies conducted in Ho Chi Minh City, Vietnam [13,14]. In contrast, other studies have shown a higher incidence in males [15-17]. This discrepancy may be related to differences in study setting, patient selection, and disease severity at admission. The Hospital for Tropical Diseases is a tertiary-level hospital within the three-tier COVID-19 treatment model established by the Ministry of Health of Vietnam; therefore, admitted patients typically had more severe disease. The study population was also predominantly older. This finding is clinically relevant because older age has consistently been associated with worse outcomes and higher mortality in patients with COVID-19 [18].

COVID-19 vaccination is crucial for primary prevention in individuals with T2DM [19]. In this study, the documented vaccination rate was relatively low, although interpretation should be cautious because vaccination status was unavailable in a substantial proportion of patients, and detailed information regarding partial versus full vaccination was not consistently recorded. In addition, the study period (June 2021 to September 2022) corresponded to the early phases of community vaccination implementation in Vietnam. Although elderly individuals and those with underlying conditions were prioritized, limited mobility during periods of social distancing and concerns regarding vaccine safety may have contributed to lower vaccine uptake. From a clinical perspective, this finding highlights the importance of improving vaccine accessibility and acceptance in high-risk groups.

Most patients in this study presented with typical respiratory manifestations, particularly cough, dyspnea, and fever. Altered mental status was observed in a subset of patients and may indicate more severe systemic illness. Previous studies have shown that altered mental status is associated with a higher risk of mortality in patients with COVID-19 [20]. This suggests that neurological status at admission should be carefully assessed in patients with COVID-19 and T2DM.

A substantial proportion of patients experienced disease progression during hospitalization. Cytokine storm may partly explain this clinical deterioration. In patients with T2DM, hyperglycemia may enhance cytokine synthesis and release, thereby amplifying the inflammatory response [21]. In this study, cytokine storm was a frequent complication. These findings are consistent with previous reports showing that patients with T2DM may develop a more pronounced inflammatory response, longer hospitalization, higher ICU admission rates, and a greater need for mechanical ventilation than non-diabetic patients [21]. The relatively severe clinical profile observed in this study may also be explained by the tertiary referral setting, which likely received a higher proportion of severe and critical cases.

Laboratory characteristics

Poor glycemic control was a prominent feature in this study. Elevated blood glucose levels were observed both at admission and during hospitalization, reflecting a combination of pre-existing metabolic dysregulation and acute stress responses associated with severe infection.

The mean HbA1c level suggested suboptimal glycemic control prior to admission. However, interpretation of HbA1c findings should be made cautiously because HbA1c was selectively performed rather than routinely applied to all patients, which may have introduced selection bias. From a clinical perspective, these findings underscore the importance of close glucose monitoring and appropriate glycemic management in hospitalized patients with COVID-19 and T2DM, as metabolic disturbances may complicate clinical management and may also reflect overall disease severity.

Microbiological characteristics

The microbiological findings in this study are broadly consistent with previous evidence showing that bacterial and fungal superinfections are common in hospitalized patients with COVID-19. In a systematic review of 118 studies, the rate of bacterial co-infection identified at the time of COVID-19 diagnosis was 8%, whereas bacterial superinfection acquired during the course of illness occurred in 20% of cases [22]. *Klebsiella pneumoniae*, *Streptococcus pneumoniae*, *Staphylococcus aureus*, and *Acinetobacter spp*. were among the most common pathogens associated with superinfection [22]. A meta-analysis of 22 studies also reported a superinfection rate of 16%, with commonly isolated microorganisms including *Pseudomonas aeruginosa*, *Escherichia coli*, *Acinetobacter baumannii*, *Haemophilus influenzae*, and invasive pulmonary aspergillosis [23]. In this study, *Acinetobacter baumannii*, *Klebsiella pneumoniae*, *Pseudomonas aeruginosa*, methicillin-resistant *Staphylococcus aureus*, *Enterococcus faecium*, *Candida albicans*, and *Candida tropicalis* were among the predominant isolates. These findings suggest that hospital-acquired infections and superinfections contributed substantially to the clinical burden in this population.

Treatment characteristics

A high proportion of patients in this study received antibiotics. This likely reflects the severity of illness at admission and the frequency of hospital-acquired infections during hospitalization. Previous studies have shown that prior continuous antibiotic exposure is associated with more severe COVID-19 outcomes, including increased hospitalization and mortality [24]. In addition, bacterial pathogens isolated from respiratory tract infections in patients with COVID-19 may exhibit substantial antimicrobial resistance [24]. However, because the timing of microbiological culture collection relative to antibiotic initiation was not consistently documented, interpretation of microbiological findings should be made cautiously. These findings underscore the importance of appropriate antibiotic use and antimicrobial stewardship.

Anticoagulant therapy was administered in most patients, reflecting the predominance of severe illness and underlying comorbidities in the study population. Anticoagulation plays an important role in the management of COVID-19-associated hypercoagulability, which may contribute to neurological and cardiovascular complications [25]. Most patients also received corticosteroids, which is consistent with their use in severe COVID-19 and in patients with marked inflammatory responses. Antiviral therapy was administered in a proportion of patients during hospitalization.

Antidiabetic medications were administered in most patients, mainly because of marked hyperglycemia, poor glycemic control, and hyperglycemia-related complications. This finding highlights the importance of glycemic management as an integral component of COVID-19 care in patients with T2DM. Disruptions in routine care during the pandemic may also have contributed to delayed diagnosis and poor metabolic control, thereby increasing the risk of adverse outcomes.

Clinical outcomes

The duration of hospitalization in this study was prolonged, and a considerable proportion of patients required ICU care and respiratory support. The in-hospital mortality was substantial. ARDS, sepsis or septic shock, ventilator-associated pneumonia, and urinary tract infection were among the most frequent complications, whereas diabetic ketoacidosis, hyperosmolar hyperglycemic state, and myocardial infarction were less common.

These findings indicate that hospitalized patients with COVID-19 and T2DM carried a high clinical burden and were vulnerable to both respiratory and non-respiratory complications. However, because this was a descriptive study conducted in a tertiary referral hospital without a non-diabetic comparison group, these findings should be interpreted within the context of the study design and should not be used to infer independent causal associations.

Limitations

This study provides real-world data from a tertiary infectious disease hospital during the COVID-19 pandemic in Vietnam and offers a relatively comprehensive description of the clinical manifestations, laboratory findings, imaging features, microbiological characteristics, treatment patterns, and outcomes of hospitalized patients with COVID-19 and T2DM, including both previously diagnosed and newly identified cases during hospitalization based on HbA1c.

However, several limitations should be considered. First, because of the retrospective descriptive design based on medical records, some variables were missing or incompletely documented, which may have introduced information bias. HbA1c testing was not routinely performed for all patients and was mainly requested based on clinical judgment, which may have led to selection bias and underestimation of newly identified T2DM. In addition, vaccination status was unavailable in a substantial proportion of patients, and detailed information regarding partial versus full vaccination was not consistently recorded, which may limit the interpretation of vaccine-related findings.

Second, because this study was conducted at a tertiary referral hospital, more severe and critical cases were likely overrepresented. This may have contributed to higher observed rates of complications, oxygen requirement, ICU admission, and mortality, and may limit the generalizability of the findings to the broader population of patients with COVID-19 and T2DM.

Third, treatment strategies for COVID-19 evolved during the study period (June 2021 to September 2022), and heterogeneity in the use of corticosteroids, anticoagulants, antibiotics, respiratory support, and glycemic management may have influenced clinical outcomes. In addition, the timing of microbiological culture collection relative to antibiotic initiation was not consistently documented, which may affect the interpretation of microbiological findings and antibiotic exposure.

Finally, the absence of a non-diabetic comparison group and the lack of comparative or multivariable analyses limited the ability to assess independent predictors or causal associations related to mortality and disease progression. Future prospective multicenter studies with larger sample sizes, systematic HbA1c testing, and inferential analyses are needed to better clarify the role of glycemic status and diabetes-related factors in COVID-19 outcomes.

## Conclusions

Hospitalized patients with COVID-19 and T2DM in this study had a high clinical burden, characterized by frequent oxygen requirement, substantial in-hospital complications, prolonged hospitalization, and considerable mortality. Poor glycemic control was also commonly observed during hospitalization. These findings support the importance of close clinical monitoring, appropriate glycemic management, and early recognition of complications in this vulnerable population.
